# Cerebral Hemodynamic Changes After Revascularization in Patients With Hemorrhagic Moyamoya Disease

**DOI:** 10.3389/fneur.2020.00072

**Published:** 2020-02-11

**Authors:** Kaijiang Kang, Ning Ma, Jinxin Li, Yuan Shen, Weibin Gu, Guofeng Ma, Dong Zhang, Xingquan Zhao

**Affiliations:** ^1^Department of Neurology, Beijing Tiantan Hospital, Capital Medical University, Beijing, China; ^2^China National Clinical Research Center for Neurological Diseases, Center of Stroke, Beijing Institute for Brain Disorders, Beijing, China; ^3^Department of Interventional Neuroradiology, Beijing Tiantan Hospital, Capital Medical University, Beijing, China; ^4^Department of Radiology, Beijing Tiantan Hospital, Capital Medical University, Beijing, China; ^5^Department of Neurosurgery, Beijing Tiantan Hospital, Capital Medical University, Beijing, China

**Keywords:** hemodynamics, intracranial hemorrhage, moyamoya disease, perfusion, revascularization

## Abstract

**Objective:** To explore the cerebral hemodynamic changes after revascularization in patients with hemorrhagic moyamoya disease (MMD).

**Materials and Methods:** We retrospectively included 57 hemorrhagic MMD patients in a high-volume stroke center from January 2016 to December 2018. All subjects were evaluated with whole-brain CT perfusion (CTP) before and after surgical revascularization. Absolute and relative CTP values in the regions of cortical middle cerebral artery territory (CMT) and deep brain area (DBA) of hemorrhagic hemispheres were measured. Differences between pre- and post-operative CTP values were assessed comprehensively. The patients were categorized into subgroups based on revascularization subtypes and postoperative CTP intervals.

**Results:** The relative cerebral blood volume (rCBV) in DBA and CMT significantly reduced in postoperative CTP (*P* < 0.05). The median and interquartile range of the proportion of rCBV decrease (rCBVc%) were 7.2% (2.3–13.2%). The rCBV reduction retained statistical significant in patients who received subtypes of revascularization, and in patients with variable intervals of follow-up (*P* < 0.05). There was no significant difference of rCBVc% between patients who received different revascularization and among patients with different postoperative CTP intervals (*P* > 0.05). The relative mean transit time (rMTT) and relative time to peak (rTTP) also showed downward trends, but without retainable statistical significance in stratified analysis. There was no significant change in relative cerebral blood flow (rCBF) (*P* > 0.05).

**Conclusion:** In patients with hemorrhagic MMD, the CBV appeared to decrease and be relatively stable in the chronic phase after revascularization, with varying degrees of MTT and TTP shortening. However, there was no significant change in CBF.

## Introduction

Moyamoya disease (MMD) is a chronic cerebrovascular disease, characterized by bilateral stenosis or occlusion at the terminal portion of the internal carotid artery and formation of abnormal vascular network at the base of the brain with unknown etiology, and it is called moyamoya syndrome (MMS) if it is associated with an underlying disease (1–3). In recent years, the prevalence and incidence of MMD has increased around the world, especially in Japan, Korea, and China (4–9). As the disease progresses, there may be varying degrees of cerebral ischemia or intracranial hemorrhage, and hemorrhagic stroke is one of the main factors leading to death and severe disability. Hemorrhagic MMD is most common in Asian populations, and the proportion of hemorrhagic MMD was up to 21 and 42.4% in the Hokkaido area of Japan and in Korea, respectively (10–12).

It is believed that intracranial hemorrhage secondary to MMD occurs mainly owing to the rupture of abnormal moyamoya vessels, dilated collateral vessels, and complicated aneurysms (13–15). Some studies have shown that revascularization surgery could promote the regression of dilated collaterals and complicated aneurysms, and then decrease the risk of rebleeding in patients with hemorrhagic MMD (16–18). However, with the regression of these collaterals, how the perfusion of brain tissue changes and whether brain tissue will face a higher risk of ischemia are still unclear. In this study, we attempted to quantitatively analyze the cerebral hemodynamic changes in hemorrhagic MMD after surgical revascularization based on CT perfusion (CTP).

## Materials and Methods

### Study Population

The study was performed according to the guidelines from the Helsinki Declaration, and it was approved by the Institutional Review Board (IRB) of the hospital. Written informed consent were obtained from all patients or their legally authorized guardian. We retrospectively and consecutively included 57 adult patients, who were diagnosed with hemorrhagic MMD, received revascularization surgery, and underwent whole-brain CTP before and after the revascularization surgery in our hospital from January 2016 to December 2018. The preoperative and postoperative CTP values of hemorrhagic hemispheres were analyzed comprehensively.

### Revascularization Surgery

The procedures were performed by experienced neurosurgeons (having performed more than 100 extra-intracranial revascularization). The neurosurgeons were instructed to choose the appropriate surgical methods based on their experience and preference as well as the conditions of individual patients. The surgical methods included direct, indirect (including encephaloduroarteriosynangiosis (EDAS) and multiple burr holes) and combined revascularization surgery.

### Clinical Data and Neuroimaging Review

Digital subtraction angiography (DSA) or (and) magnetic resonance imaging (MRI) were performed for all of the patients in our hospital to confirm the diagnosis of MMD according to the diagnostic guidelines proposed by the Ministry of Health and Welfare of Japan (19). Each hemisphere was analyzed separately and all of the hemorrhagic hemispheres were confirmed by computed tomography (CT). For bilateral intraventricular hemorrhage (IVH) or subarachnoid hemorrhage (SAH), the side with larger hemorrhage volume were regarded as hemorrhagic hemisphere, and those indistinguishable hemispheres were excluded.

All the images of CTP were interpreted by two experienced neuroradiologists independently, and CTP interpretation training program was performed for both of them to ensure the consistency of interpreting standard. The differences between their assessments were resolved by a third senior neurologist (categorical data), or we took the average of their data (quantitative data) to reduce the impact of subjective factors.

### CT Scanning and Processing of CTP Data

The imaging protocol includes whole brain CTP before and after revascularization surgery. CTP studies were performed in the transverse plane by using a 256-slice axial CT scanner (GE Revolution CT) before and after surgical revascularization for all subjects. A 50-ml bolus of contrast media (Omnipaque, 350 mg I/ml; GE Healthcare, Shanghai, CN) was administered into an antecubital vein by using a power injector (Ulrich Injection System; Ulrich GMBH & CO. KG, Germany) with an injection rate of 5 ml/s. CT scanning was initiated 5 s after the start of the injection with the following acquisition parameters: 80 kV tube voltage, 150 mA, 5 mm slice thickness, 256*0.625 mm collimation, 0.5 s rotation time, 2.0 s cycle time, 25 cm field-of-view (FOV), 512*512 image matrix size, 32 slices. A total of 512 slices were obtained with a scan time of about 40 s and 160 mm scan length. Brain standard reconstruction was performed with the CT system.

CTP source data were transferred to a designated workstation (GE Workstation aw 4.7), and the whole-brain CTP images were reconstructed from source data for analysis. The software relies on the central volume principle to calculate perfusion parameters from the time-concentration curve. The mean transit time (MTT) was calculated by a closed-form deconvolution operation from the time-concentration curve and the arterial input function (AIF). The first artery to reach peak enhancement on the time-attenuation curve was selected as the AIF. The vein with the largest area under the time-attenuation curve was selected as the venous outflow function (VOF). The cerebral blood volume (CBV) was calculated from the areas under the time-concentration curves. The cerebral blood flow (CBF) was calculated according to the following equation according to the CBV and MTT values: CBF = CBV/MTT. The time to peak (TTP) refers to the length of time for brain tissue to reach enhancement of peak density. Large vessels were automatically excluded via the brain perfusion software.

### Regions of Interest (ROIs) Selection

**Figure 1:**
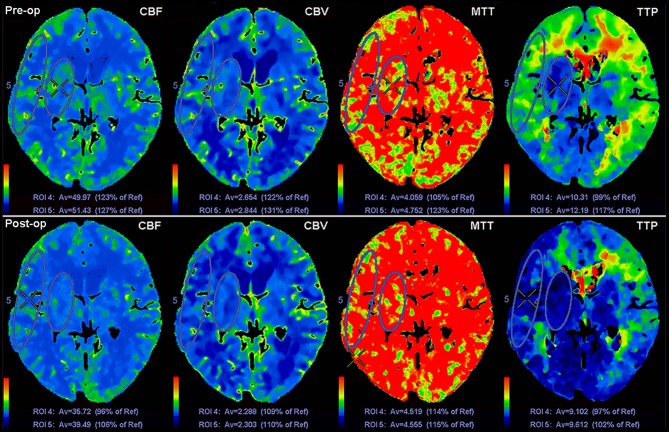
Schematic diagram of ROI drawing in the region of DBA and CMT, and changes of CTP values before and after revascularization surgery.

Two experienced neuroradiologists independently drew standardized elliptical regions of interest (ROIs) manually on the basal ganglia (BG) section level of the reference CT image over the cortical middle cerebral artery (MCA) territory (CMT) and deep brain area (DBA) (Figure 1). If there were previous hemorrhagic or infarcted lesions in the area of interest, we have made some adjustments to the size and shape of the ROIs to avoid the previous lesions. But in the same patient, an attempt was made to draw almost same ROIs in same areas before and after the revascularization. In patients with MMD, anterior cerebral arteries such as ACA and MCA might be occluded and continuously progress, so it is not reasonable to use anterior mirror ROIs as reference area. Therefore, we selected pons (basilar artery territory) as reference area manually in this study. The CTP absolute values of hemorrhagic hemispheres in the region of CMT, DBA, and reference regions (pons) in functional maps were measured (Figure 1). For each ROI, CBF, CBV, MTT, and TTP values were automatically calculated by the software. The relative cerebral blood flow (rCBF), relative cerebral blood volume (rCBV), relative mean transit time (rMTT), and relative time to peak (rTTP) were obtained as follow: relative CTP values = absolute CTP values in CMT or DBA/absolute CTP values in pons. The proportion of relative CTP value change (rCTPc%) was defined as the mean value of rCTPc% in DBA and CMT, and it was obtained as follow: rCTPc% = (postoperative rCTP value—preoperative rCTP value)/postoperative rCTP value.

### Angiographic Evaluation

CT angiography was also reconstructed from the postoperative CTP source data to evaluate the angiographic outcome of revascularization. Based on the results of CT angiography, development of postoperative revascularization collaterals was defined as three levels [similar to the system described by Matsushima et al. (20)]: good revascularization (area perfused by postoperative revascularization collaterals more than 2/3 of the middle cerebral artery (MCA) distribution), moderate revascularization (between 1/3 and 2/3 of the MCA distribution), and poor revascularization (<1/3 of the MCA distribution).

### Statistical Analysis

The statistical analysis was performed using a commercial statistical software package (SPSS for Windows, version 22.0, IBM-SPSS, Chicago, IL, US). For CTP parameters, the mean of ROI values on each hemorrhagic hemispheres and pons were calculated before and after the surgery. Normal distribution data were expressed as mean ± SD while skew distribution data were expressed as median (IQR). Differences in pre- and postoperative CTP values were assessed using paired *t*-test (normal distribution) or non-parametric test (skew distribution). Differences of *P* < 0.05 were considered statistically significant for two-tailed tests.

## Results

Fifty-seven hemorrhagic MMD adults (58 hemorrhagic hemispheres) were included in this study and each hemisphere was analyzed separately. The mean age was 39.6 ± 9.7 years old, and males accounted for 29.3% of the patients. Of the 58 hemorrhagic hemispheres, 23 hemispheres (39.7%) received indirect revascularization, and other 35 hemispheres (60.3%) received direct or combined revascularization. The median of duration from first intracranial hemorrhage to preoperative CTP, from preoperative CTP to revascularization, from revascularization to postoperative CTP were 6.5 months (IQR, 3–13), 7 days (IQR, 4–23), and 6.5 months (IQR, 4–11) respectively. The demographic and clinical information of patients were shown in Table 1. The included patients were followed up for 6–34 months with a median duration of 12 months (no patient was lost to follow-up), and postoperative rebleeding occurred only in one hemisphere (1.7%).

**Table 1:** Demographic and clinical information of the included patients.

**Variable**	**Value**
Number of patients	57
Number of hemispheres	58
Male, *n* (%)	17 (29.3%)
Age, mean ± SD	39.6 ± 9.7
**Risk factors**	
Hypertension, *n* (%)	13 (22.4%)
Diabetes mellitus, *n* (%)	0 (0%)
Hyperlipidemia, *n* (%)	1 (1.7%)
Smoking, *n* (%)	6 (10.3%)
**Types of hemorrhage**	
IVH, *n* (%)	16 (27.6%)
ICH+IVH, *n* (%)	24 (41.4%)
ICH, *n* (%)	12 (20.7%)
SAH, *n* (%)	6 (10.3%)
**Frequency of hemorrhage**	
1, n (%)	49 (84.5%)
2, n (%)	8 (13.8%)
3, n (%)	1 (1.7%)
**Suzuki stage**	
Suzuki 1–2	1 (2%)
Suzuki 3–4	39 (79.6%)
Suzuki 5–6	8 (18.4)
Duration from first hemorrhage to preoperative CTP (months), median (IQR)	6.5 (3–13)
Duration from preoperative CTP to revascularization (days), median (IQR)	7 (4–23)
Duration from revascularization to postoperative CTP (months), median (IQR)	6.5 (4–11)

*IVH, intraventricular hemorrhage; ICH, intracerebral hemorrhage; SAH, subarachnoid hemorrhage; CTP, CT perfusion*.

Perioperative complications had been evaluated, and no serious complications occurred in the 58 hemispheres included in this study. The perioperative neurological complications occurred in 17.2% (10/58) of the hemispheres, including transient ischemic attacks (*n* = 6), minor ischemic stroke (*n* = 2), hyperperfusion syndrome without hemorrhage (*n* = 1), and seizures (*n* = 1). These complications resulted mainly from transient hemodynamic changes after the revascularization, and may not have much impact on the long-term cerebral hemodynamics.

The results of CT angiography showed that the good, moderate and poor revascularization collaterals were observed in 32 (55.2%), 20 (34.5%), and 6 patients (10.3%), respectively. The proportion of good, moderate and poor revascularization collaterals in hemispheres with direct or combined revascularization were 71.4% (25/35), 25.7% (9/35), and 2.9% (1/35), respectively. The proportion of good, moderate and poor revascularization collaterals in hemispheres with indirect revascularization were 30.4% (7/23), 47.8% (11/23), and 21.7% (5/23), respectively.

In the comparison of pre- and postoperative absolute CTP values, the median of CBV decreased significantly after revascularization, from 2.64 to 2.49 ml·100 g^−1^ in DBA (*P* = 0.018), and from 3.22 to 3.02 ml·100 g^−1^ in CMT (*P* < 0.001). The mean of CBF in DBA decreased from 48.77 ± 11.10 to 44.82 ± 9.94 ml·100 g^−1^·min^−1^ (*P* = 0.001) and the median of TTP in CMT decreased from 11.93 to 10.82 s (*P* = 0.001). However, there was no significant change in MTT and TTP in DBA, or CBF and MTT in CMT (*P* > 0.05) (Table 2). In addition, there was no significant difference in the absolute values of CBF, CBV, MTT, or TTP in pons (reference area) between preoperative and postoperative CTP (*P* > 0.05).

**Table 2:** Pre- and postoperative comparison of CTP values of hemorrhagic hemispheres in the region of DBA and CMT.

**CTP values**	**DBA**			**CMT**		
	**Pre-operation**	**Post-operation**	***P*-value**	**Pre-operation**	**Post-operation**	***P*-value**
CBF (ml·100 g^−1^·min^−1^)	48.77 ± 11.10	44.82 ± 9.94	0.001	53.28 (48.32–63.25)	51.83 (40.06–60.53)	0.157
CBV (ml·100 g^−1^)	2.64 (2.32–3.17)	2.49 (2.36–2.80)	0.018	3.22 (2.74–3.63)	3.02 (2.58–3.23)	<0.001
MTT(s)	4.38 ± 1.31	4.34 ± 1.10	0.825	4.35 (3.74–5.52)	4.18 (3.50–5.24)	0.377
TTP(s)	10.75 ± 1.48	10.39 ± 1.68	0.110	11.93 (10.78–12.80)	10.82 (9.95–12.08)	0.001
rCBF	1.07 (0.94–1.17)	1.03 (0.92–1.12)	0.126	1.19 ± 0.35	1.18 ± 0.25	0.777
rCBV	1.20 (1.08–1.31)	1.09 (1.02–1.22)	<0.001	1.44 ± 0.25	1.29 ± 0.20	<0.001
rMTT	1.20 (1.02–1.47)	1.18 (1.00–1.38)	0.158	1.29 (1.09–1.59)	1.20 (1.00–1.36)	0.022
rTTP	1.05 ± 0.07	1.04 ± 0.06	0.010	1.15 (1.08–1.25)	1.08 (1.04–1.15)	<0.001

*DBA, deep brain area; CMT, cortical MCA territory; CBF, cerebral blood flow; CBV, cerebral blood volume; MTT, mean transit time; TTP, time to peak; rCBF, relative cerebral blood flow; rCBV, relative cerebral blood volume; rMTT, relative mean transit time; rTTP, relative time to peak. Normal distribution data were expressed as mean ± SD, while skew distribution data were expressed as median (IQR)*.

**Figure 2:**
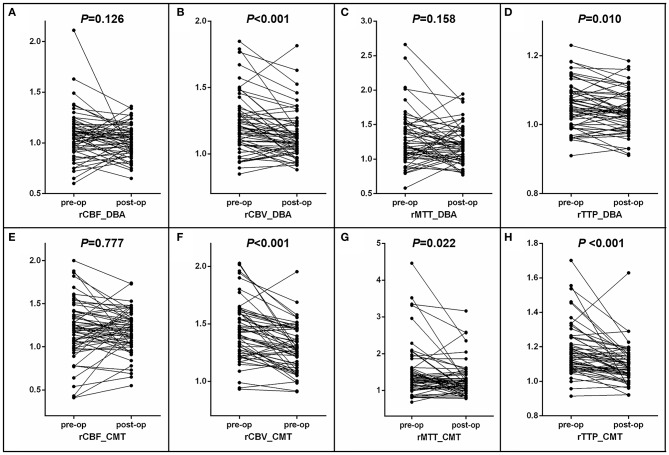
Pre- and postoperative comparison of CTP values of hemorrhagic hemispheres in the region of DBA and CMT. **(A–D)** Comparison of CTP values of hemorrhagic hemispheres in the region of DBA. **(E–H)** Comparison of CTP values of hemorrhagic hemispheres in the region of CMT.

**Figure 3:**
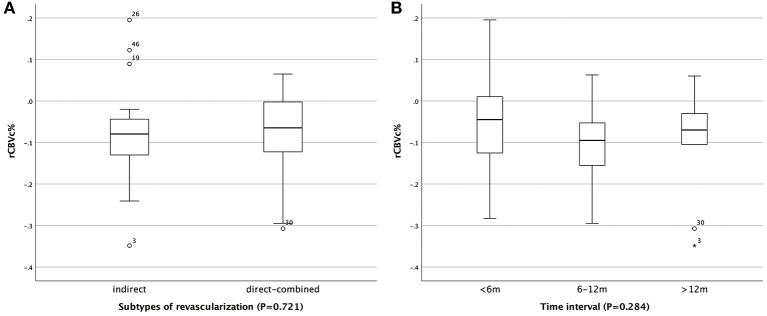
Comparison of rCBVc% in patients who received different revascularization and among patients with different postoperative CTP intervals. **(A)** Comparison of rCBVc% in patients who received subtypes of revascularization. **(B)** Comparison of rCBVc% among patients with different postoperative CTP intervals.

In the comparison of pre- and postoperative relative CTP values, there were significant reduction of rCBV in DBA and CMT (*P* < 0.001) (Table 2, Figure 2). The median and interquartile range of the proportion of rCBV decrease (rCBVc%) were 7.2% (2.3–13.2%). In stratified analysis, the rCBV reduction retained statistical significance in patients who received subtypes of revascularization (23 received indirect revascularization and 35 received direct or combined revascularization) (Table 3), and in patients with varying postoperative CTP intervals (Table 4) (*P* < 0.05). There was no significant difference of rCBVc% between patients who received indirect revascularization and those who received direct or combined revascularizarion (Figure 3). There was also no significant difference of rCBVc% among patients with <6 months, 6–12 months, or >12 months postoperative CTP (Figure 3).

**Table 3:** Pre- and postoperative comparison of CTP values of hemorrhagic hemispheres in the region of DBA and CMT among different types of revascularization.

**Revascularization**	**CTP values**	**DBA**			**CMT**		
		**Pre-operation**	**Post-operation**	***P*-value**	**Pre-operation**	**Post-operation**	***P*-value**
Indirect (*n* = 23)	rCBF	1.00 (0.93–1.18)	1.03 (0.90–1.18)	0.855	1.15 ± 0.36	1.13 ± 0.28	0.734
	rCBV	1.23 (1.15–1.48)	1.14 (1.07–1.32)	0.006	1.47 ± 0.28	1.33 ± 0.25	0.003
	rMTT	1.34 ± 0.32	1.23 ± 0.24	0.125	1.33 (1.13–1.60)	1.26 (1.04–1.57)	0.136
	rTTP	1.07 ± 0.06	1.05 ± 0.05	0.009	1.18 (1.11–1.26)	1.11 (1.08–1.18)	0.015
Direct or Combined (*n* = 35)	rCBF	1.06 ± 0.19	1.01 ± 0.14	0.102	1.22 ± 0.34	1.22 ± 0.22	0.920
	rCBV	1.19 ± 0.20	1.10 ± 0.14	0.001	1.42 ± 0.24	1.26 ± 0.17	<0.001
	rMTT	1.18 (1.01–1.40)	1.14 (0.99–1.28)	0.670	1.23 (1.03–1.57)	1.12 (0.99–1.29)	0.098
	rTTP	1.03 (0.99–1.08)	1.03 (0.98–1.06)	0.190	1.11 (1.07–1.20)	1.05 (1.03–1.12)	<0.001

*DBA, deep brain area; CMT, cortical MCA territory; CBF, cerebral blood flow; CBV, cerebral blood volume; MTT, mean transit time; TTP, time to peak; rCBF, relative cerebral blood flow; rCBV, relative cerebral blood volume; rMTT, relative mean transit time; rTTP, relative time to peak. Normal distribution data were expressed as mean ± SD, while skew distribution data were expressed as median (IQR)*.

**Table 4:** Pre- and postoperative comparison of CTP values of hemorrhagic hemispheres in the region of DBA and CMT among patients with different postoperative CTP intervals.

**Post-operative CTP intervals**	**CTP values**	**DBA**			**CMT**		
		**Pre-operation**	**Post-operation**	***P*-value**	**Pre-operation**	**Post-operation**	***P*-value**
<6 months (*n* = 26)	rCBF	1.06 ± 0.21	0.99 ± 0.16	0.073	1.17 ± 0.30	1.14 ± 0.25	0.650
	rCBV	1.19 (1.08–1.31)	1.10 (1.03–1.28)	0.034	1.42 ± 0.30	1.31 ± 0.23	0.017
	rMTT	1.20 (1.01–1.51)	1.22 (1.11–1.46)	0.568	1.28 (1.10–1.65)	1.25 (1.01–1.53)	0.585
	rTTP	1.04 (1.00–1.08)	1.03 (1.00–1.09)	0.970	1.13 (1.08–1.25)	1.10 (1.06–1.16)	0.004
6–12 months (*n* = 22)	rCBF	1.08 (0.94–1.25)	1.09 (0.95–1.13)	0.833	1.20 ± 0.42	1.20 ± 0.28	0.995
	rCBV	1.20 (1.09–1.37)	1.10 (0.98–1.22)	0.001	1.47 ± 0.20	1.28 ± 0.20	<0.001
	rMTT	1.18 (1.03–1.41)	1.16 (0.94–1.35)	0.046	1.26 (1.09–1.73)	1.13 (1.00–1.29)	0.022
	rTTP	1.05 ± 0.06	1.03 ± 0.06	0.002	1.16 (1.09–1.27)	1.06 (1.03–1.14)	0.002
>12 months (*n* = 10)	rCBF	1.04 ± 0.15	1.04 ± 0.18	0.902	1.23 ± 0.32	1.23 ± 0.13	0.990
	rCBV	1.20 (0.99–1.26)	1.07 (1.03–1.15)	0.114	1.45 ± 0.25	1.26 ± 0.16	0.032
	rMTT	1.32 (0.99–1.52)	1.07 (0.92–1.21)	0.241	1.33 (1.02–1.53)	1.12 (0.85–1.32)	0.285
	rTTP	1.06 ± 0.08	1.03 ± 0.07	0.024	1.15 ± 0.17	1.07 ± 0.09	0.158

*rCBF, relative cerebral blood flow; rCBV, relative cerebral blood volume; rMTT, relative mean transit time; rTTP, relative time to peak. P < 0.05 was considered statistically significant. Normal distribution data were expressed as mean ± SD while skew distribution of measurement data were expressed as median (IQR)*.

The relative mean transit time (rMTT) and relative time to peak (rTTP) also showed downward trends in DBA and CMT, which did not retain statistically significant in the patients who received different types of revascularization, or in the patients with different postoperative CTP intervals. In addition, there was no retainable significant change in rCBF whether in DBA or CMT (Tables 2–4, Figure 2).

## Discussions

Hemorrhagic stroke is one of the main factors leading to worse prognosis, and occurs mainly owing to the rupture of abnormally dilated and fragile vessels and complicated aneurysms (13–15). Revascularization has been aimed at improving cerebral hemodynamics and then preventing recurrent strokes in patients with MMD. It is generally believed that surgical revascularization could increase cerebral blood flow and then alleviate the dilation of collateral vessels or complicated aneurysms, which may contribute to decreasing the risk of rebleeding in patients with hemorrhagic MMD (13, 17, 18, 21). However, with the establishment of artificial collateral vessels and the regression of original collateral vessels, how the cerebral hemodynamics changes in hemorrhagic MMD after surgical revascularization is still unclear.

CTP, as a reliable and readily accessible technique method, has been widely used in cerebral hemodynamic evaluation of MMD in clinical practice (22–25). In this study, we found that in adult patients with hemorrhagic MMD, the most significant hemodynamic change after revascularization surgery was CBV reduction, accompanied by varying degrees of MTT and TTP shortening. However, there was no significant change in CBF.

Previous studies suggested that in patients with MMD, the CBF and CBV significantly increased after surgical revascularization (24, 26–28). However, in previous studies, the patients included were mainly ischemic MMD, of which hemodynamic characteristics may be different from those of hemorrhagic MMD. Also, some studies investigated just the acute changes after revascularization, which may be complicated with postoperative hyperperfusion (28). In addition, the selection of a reference ROI is very important. In most studies, contralateral mirroring areas has been selected as reference ROI, which is unreasonable because anterior cerebral arteries such as anterior cerebral artery (ACA) and middle cerebral artery (MCA) might be occluded or be occluding with the progress of moyamoya disease, and the CTP parameters may be also changing accordingly (24, 26). In this study, we selected pons (basilar artery territory) as reference area manually, and found that in patients with hemorrhagic MMD, the CBF did not increase, and the CBV even decreased significantly after revascularization either in DBA or CMT, which was significantly different from previous studies. Decrease in CBV means reduction of vascular bed area and retraction of abnormal moyamoya vessels and dilated collateral vessels, which may contribute to the reduction of the pressure load of the vascular wall, and then reduce the risk of rebleeding. Theoretically, MTT and TTP should be decreased in CMT area after successful revascularization. In this study however, MTT and TTP presented shortening tendencies, but did not show consistent changes. From the point of our view, it may be related to the following factors. First, there was no consistent statistical significance, possibly because the sample size of this study was relatively small. Second, we did not exclude patients with poor revascularization, which were more frequent in the indirect revascularization group. We also noticed that the MTT and TTP in the direct or combined revascularization group were more decreased in CMT than that in the indirect revascularization group. Therefore, we speculate that in patients with hemorrhagic MMD, the risk of rebleeding decreases after revascularization surgery, probably not because of increased cerebral blood flow, but because the blood flow pattern has changed, from a slow and stagnant status to a pattern of fast and efficient blood flow.

Currently, for patients with MMD (whether ischemic or hemorrhagic type) who have received surgical revascularization, the hemodynamic changes that we are most concerned about is still CBF. However, from the results of this study, for patients with hemorrhagic MMD, the most significant hemodynamic change after revascularization surgery is CBV. However, in the present study, most patients have a good outcome during the follow-up period, and postoperative rebleeding occurred only in one hemisphere. Therefore, we cannot conclude whether decrease in CBV is associated with good outcome or reduced risk of rebleeding, and studies with larger sample size and longer follow-up intervals are needed to confirm whether CBV can be a neuroradiological marker for evaluating the outcome of revascularization surgery and the risk of rebleeding in patients with hemorrhagic MMD.

Potential limitations of our studies should be mentioned. First, all of the patients in this study were enrolled from a single center, and the surgical methods were not randomly assigned, so potential selection bias may be inevitable. Second, ROIs in this study were drawn manually and only parts of the MCA distribution area were evaluated. It is practically challenging to draw ROIs in a completely consistent area before and after the revascularization. However, we had tried our best to draw almost same ROIs in same areas before and after the revascularization. Third, the retrospective nature of the study necessitates further prospective cohorts or randomized studies to confirm our conclusions.

## Conclusion

In patients with hemorrhagic MMD, the CBV appeared to decrease and be relatively stable in the chronic phase after revascularization, with varying degrees of MTT and TTP shortening. However, there was no significant change in CBF.

## Data Availability Statement

The raw data supporting the conclusions of this article will be made available by the authors, without undue reservation, to any qualified researcher.

## Ethics Statement

The study was performed according to the guidelines from the Helsinki Declaration, and it was approved by the Institutional Review Board (IRB) of the hospital. Written informed consent were obtained from all patients or their legally authorized guardian.

## Author Contributions

KK contributed to the conception/design of the study, the acquisition, analysis and interpretation of data, drafting of the manuscript, and final approval of the version to be published. NM, JL, and YS were responsible for acquisition of clinical data, data analysis, and final approval of the version to be published. WG and GM were responsible for acquisition of data and final approval of the version to be published. XZ and DZ were responsible for conception/design of the study, revision of the work, final approval of the version to be published, and agreement to be accountable for all aspects of the work.

### Conflict of Interest

The authors declare that the research was conducted in the absence of any commercial or financial relationships that could be construed as a potential conflict of interest.

## References

[1] Scott RM Smith ER. Moyamoya disease and moyamoya syndrome. N Engl J Med. (2009) 360:1226–37. 10.1056/NEJMra0804622 19297575

[2] Kuroda S Houkin K. Moyamoya disease: current concepts and future perspectives. Lancet Neurol. (2008) 7:1056–66. 10.1016/S1474-4422(08)70240-0 18940695

[3] Phi JHW Wang KC Lee JY Kim SK. moyamoya syndrome: a window of moyamoya disease. J Korean Neurosurg Soc. (2015) 57:408–14. 10.3340/jkns.2015.57.6.408 26180607 PMC4502236

[4] Kim JS. Moyamoya disease: epidemiology, clinical features, and diagnosis. J Stroke. (2016) 18:2–11. 10.5853/jos.2015.01627 26846755 PMC4747069

[5] Kuriyama S Kusaka Y Fujimura M Wakai K Tamakoshi A Hashimoto S. Prevalence and clinicoepidemiological features of moyamoya disease in Japan: findings from a nationwide epidemiological survey. Stroke J Cereb Circul. (2008) 39:42–7. 10.1161/STROKEAHA.107.490714 18048855

[6] Kim T Lee H Bang JS Kwon OK Hwang G Oh CW. Epidemiology of moyamoya disease in korea: based on national health insurance service data. J Korean Neurosurg Soc. (2015) 57:390–5. 10.3340/jkns.2015.57.6.390 26180604 PMC4502233

[7] Duan L Bao XY Yang WZ Shi WC Li DS Zhang ZS. Moyamoya disease in China: its clinical features and outcomes. Stroke J Cereb Circul. (2012) 43:56–60. 10.1161/STROKEAHA.111.621300 22020027

[8] Bao XY Wang QN Zhang Y Zhang Q Li DS Yang WZ. Epidemiology of moyamoya disease in China: single-center, population-based study. World Neurosurg. (2019) 122:e917–e23. 10.1016/j.wneu.2018.10.175 30404059

[9] Chen PC Yang SH Chien KL Tsai IJ Kuo MF. Epidemiology of moyamoya disease in Taiwan: a nationwide population-based study. Stroke J Cereb Circul. (2014) 45:1258–63. 10.1161/STROKEAHA.113.004160 24676775

[10] Baba T Houkin K Kuroda S. Novel epidemiological features of moyamoya disease. J Neurol Neurosurg Psychiatr. (2008) 79:900–4. 10.1136/jnnp.2007.130666 18077479

[11] Ikezaki K Han DH Kawano T Kinukawa N Fukui M. A clinical comparison of definite moyamoya disease between South Korea and Japan. Stroke J Cereb Circul. (1997) 28:2513–7. 10.1161/01.STR.28.12.2513 9412642

[12] Hori S Kashiwazaki D Yamamoto S Acker G Czabanka M Akioka N. Impact of interethnic difference of collateral angioarchitectures on prevalence of hemorrhagic stroke in moyamoya disease. Neurosurgery. (2018) 85:134–146. 10.1093/neuros/nyy236 29889273

[13] Liu P Han C Li DS Lv XL Li YX Duan L Hemorrhagic moyamoya disease in children: clinical, angiographic features, and long-term surgical outcome. Stroke J Cereb Circul. (2016) 47:240–3. 10.1161/STROKEAHA.115.010512 26534975

[14] Funaki T Takahashi JC Houkin K Kuroda S Takeuchi S Fujimura M. High rebleeding risk associated with choroidal collateral vessels in hemorrhagic moyamoya disease: analysis of a nonsurgical cohort in the Japan Adult Moyamoya Trial. J Neurosurg. (2019) 130:337–673. 10.3171/2017.9.JNS17576 29498573

[15] Morioka M Hamada J Kawano T Todaka T Yano S Kai Y. Angiographic dilatation and branch extension of the anterior choroidal and posterior communicating arteries are predictors of hemorrhage in adult moyamoya patients. Stroke J Cereb Circul. (2003) 34:90–5. 10.1161/01.STR.0000047120.67507.0D 12511756

[16] Wang QN Bao XY Zhang Y Zhang Q Li DS Duan L Encephaloduroarteriosynangiosis for hemorrhagic moyamoya disease: long-term outcome of a consecutive series of 95 adult patients from a single center. J Neurosurg. (2018) 130:1–8. 10.3171/2017.12.JNS172246 29999465

[17] Liu X Zhang D Shuo W Zhao Y Wang R Zhao J. Long term outcome after conservative and surgical treatment of haemorrhagic moyamoya disease. J Neurol Neurosurg Psychiatr. (2013) 84:258–65. 10.1136/jnnp-2012-302236 23012444

[18] Jiang H Yang H Ni W Lei Y Su J Gu Y. Long-term outcomes after combined revascularization surgery in adult hemorrhagic moyamoya disease. World Neurosurg. (2018) 116:e1032–41. 10.1016/j.wneu.2018.05.153 29859362

[19] Research committee on the pathology and treatment of spontaneous occlusion of the circle of Willis Guidelines for diagnosis and treatment of moyamoya disease (spontaneous occlusion of the circle of Willis). Neurol Med Chirurgica. (2012) 52:245–66. 10.2176/nmc.52.245 22870528

[20] Matsushima T Inoue T Suzuki SO Fujii K Fukui M Hasuo K. Surgical treatment of moyamoya disease in pediatric patients–comparison between the results of indirect and direct revascularization procedures. Neurosurgery. (1992) 31:401–5. 10.1097/00006123-199209000-00003 1407421

[21] Kang K Lu J Zhang D Li Y Wang D Liu P. Difference in cerebral circulation time between subtypes of moyamoya disease and moyamoya syndrome. Scient Rep. (2017) 7:2587. 10.1038/s41598-017-02588-1 28566764 PMC5451479

[22] Rim NJ Kim HS Shin YS Kim SY. Which CT perfusion parameter best reflects cerebrovascular reserve?: correlation of acetazolamide-challenged CT perfusion with single-photon emission CT in Moyamoya patients. AJNR Am J Neuroradiol. (2008) 29:1658–63. 10.3174/ajnr.A1229 18617583 PMC8118762

[23] Kang KH Kim HS Kim SY. Quantitative cerebrovascular reserve measured by acetazolamide-challenged dynamic CT perfusion in ischemic adult Moyamoya disease: initial experience with angiographic correlation. AJNR Am J Neuroradiol. (2008) 29:1487–93. 10.3174/ajnr.A1129 18499785 PMC8119064

[24] Zhang J Wang J Geng D Li Y Song D Gu Y. Whole-brain CT perfusion and CT angiography assessment of Moyamoya disease before and after surgical revascularization: preliminary study with 256-slice CT. PLoS ONE. (2013) 8:e57595. 10.1371/journal.pone.0057595 23451248 PMC3579776

[25] Sasagawa A Mikami T Hirano T Akiyama Y Mikuni N. Characteristics of cerebral hemodynamics assessed by CT perfusion in moyamoya disease. J Clin Neurosci. (2018) 47:183–9. 10.1016/j.jocn.2017.09.020 29056445

[26] Chen Y Xu W Guo X Shi Z Sun Z Gao L. CT perfusion assessment of Moyamoya syndrome before and after direct revascularization (superficial temporal artery to middle cerebral artery bypass). Europ Radiol. (2016) 26:254–61. 10.1007/s00330-015-3802-4 25925360

[27] Li Z Zhou P Xiong Z Ma Z Wang S Bian H. Perfusion-weighted magnetic resonance imaging used in assessing hemodynamics following superficial temporal artery-middle cerebral artery bypass in patients with Moyamoya disease. Cerebrovasc Dis. (2013) 35:455–60. 10.1159/000350197 23735877

[28] Kaku Y Iihara K Nakajima N Kataoka H Fukuda K Masuoka J. Cerebral blood flow and metabolism of hyperperfusion after cerebral revascularization in patients with moyamoya disease. J Cereb Blood Flow Metab. (2012) 32:2066–75. 10.1038/jcbfm.2012.110 22850406 PMC3493997

